# Smartphone 3D Scanning Technology and 3D Semi-Synthetic Data for Processing Infant Head Deformities Using Artificial Intelligence

**DOI:** 10.3390/s26051444

**Published:** 2026-02-25

**Authors:** Omar C. Quispe-Enriquez, José Luis Lerma

**Affiliations:** Photogrammetry and Laser Scanner Research Group (GIFLE), Department of Cartographic Engineering, Geodesy and Photogrammetry, Universitat Politècnica de València, Camino de Vera s/n, 46022 Valencia, Spain; ocquienr@doctor.upv.es

**Keywords:** mobile phone, semi-synthetic data, infant cranial deformities, artificial intelligence, machine learning, 3D point clouds processing

## Abstract

**Background**: Early assessment of cranial deformities in newborns, such as plagiocephaly, brachycephaly, dolichocephaly, turricephaly, and trigonocephaly, requires precise and non-invasive methods. **Methodology**: This study presents a methodology using a 3D scanning smartphone application to capture three-dimensional head point clouds. A total of 60 3D point cloud cases were classified according to six classes of head deformities. These 60 real 3D point clouds were expanded to 3600 semi-synthetic point clouds via controlled geometric transformations simulating realistic cranial variations. A total of 138 morphometric descriptors were extracted per class, representing spatial head features as distances from the centre of the point cloud to the head surface. These descriptors were used to train and compare three machine learning models: decision tree, random forest, and multilayer perceptron, enabling the automatic classification of six infant’s head deformities. **Results**: The machine learning models achieved high classification accuracy, with F1-scores up to 0.98, demonstrating the effectiveness of the approach. **Conclusions**: The results demonstrate the potential of combining mobile 3D sensors, image-based modelling, semi-synthetic data, and artificial intelligence to provide predictive support in clinical applications, highlighting the usefulness of low-cost portable optical sensors.

## 1. Introduction

Three-dimensional (3D) reconstruction of cranial geometry is a key aspect in paediatric biometric applications, especially when non-invasive, portable, and low-cost solutions are required. In recent years, advances in image sensors embedded in mobile devices, along with photogrammetric solutions using Structure-from-Motion (SfM) and Multi-View Stereo (MVS) techniques, have made it possible to generate dense point clouds and detailed 3D models from conventional visible (RGB) cameras, expanding the use of portable systems for geometric acquisition and modelling [1,2]. These approaches have shown that smartphones can act as platforms for image reconstruction, retrieval, and modelling applications in different scientific and clinical settings [3].

From an image-processing point of view, 3D reconstruction techniques based on optical sensors make it possible to obtain dense point clouds that describe the complete geometry of the captured object. These representations can be processed using surface reconstruction algorithms and geometric analysis, providing more robust and complete morphometric descriptors than two-dimensional (2D) methods, including photography, or manual measurements [4]. In the biometric field, this ability is especially relevant for the objective characterisation of complex anatomical structures.

One of the clinical applications where these technologies have shown high potential is the evaluation of childhood cranial deformities, such as plagiocephaly, brachycephaly or dolichocephaly. These deformities are common during the first months of life, and their early detection is essential to avoid functional and aesthetic complications. Traditionally, diagnosis has been based on physical examination and clinical history, procedures with a high subjective component and dependent on the examiner’s experience [5,6,7]. In cases of suspected craniosynostosis, clinical guidelines recommend the use of computed tomography (CT), although these techniques have relevant limitations, such as exposure to ionising radiation, high cost and, in many cases, the need for paediatric sedation, and cannot be used extensive [8,9,10,11,12].

There are currently several instrumental methods for quantifying cranial shape. Plagiocephalometry has established itself as a reproducible technique for measuring cranial asymmetry; however, its application is laborious and does not allow the full capture of the three-dimensional (3D) nature of the deformity [13,14]. Similarly, manual anthropometric measurements provide simple indices, although they have considerable interobserver variability and a limited geometric description of the skull [15,16]. In contrast, techniques based on 3D imaging methods allow an exhaustive characterisation of the cranial shape by recovering the complete geometry [4]; alternatively, deep learning-based methods also exist, although there are still restrictive limitations due to perspectivity.

In recent years, mobile applications have emerged aimed at analysing children’s cranial shape from 2D photographs acquired in a standardised overhead view, such as Skully Care and Little Angel Medical. These tools are notable for their ease of use and speed, but are limited to 2D measurements, which restricts their ability to evaluate complex deformities and does not allow the full 3D geometry of the skull to be recovered [17]. Despite the latter being able to integrate artificial intelligence from a single image, more promising results are expected using three orthogonal images for future craniosynostosis screenings [18]. Performance has been achieved in two-dimensional processing tasks [19,20,21]. To overcome these limitations, photogrammetry and 3D scanning make it possible to reconstruct complete models of the head, improving the accuracy of geometric analysis and clinical evaluation [22,23,24,25].

Smartphone-based photogrammetry is a non-invasive alternative for the 3D reconstruction of infant heads, achieving levels of precision comparable to those of medical imaging techniques in cranial morphometric analysis [23,24,26]. However, the availability of 3D clinical datasets remains limited, making it difficult to train robust machine learning and deep learning models.

To address this limitation, semi-synthetic data generation strategies have been proposed by applying controlled geometric transformations on real 3D models, preserving anatomical plausibility and expanding the available morphological variability [27,28]. In addition, generative artificial intelligence approaches make it possible to create synthetic datasets that facilitate the development of predictive models and contribute to the protection of patient privacy [29]. Advances in convolutional neural networks (CNNs) and deep learning (DL) have demonstrated high potential in automatic medical image analysis [30,31].

This paper presents a methodology that integrates image acquisition using the camera sensor integrated into smartphones, 3D reconstruction through PhotoMeDAS [32]. From the 3D models obtained, semi-synthetic data are generated, and machine learning models are trained for the classification of childhood cranial deformities. The proposed approach reduces reliance on specialised equipment, improves accessibility in resource-limited clinical settings, and provides an objective and reproducible characterisation of cranial shape.

The main contributions of this study include the presentation of a non-invasive, low-cost and easy-to-use smartphone-based scanning application for capturing infant’s head geometry; the expansion of a limited dataset of 60 real 3D point clouds to 3600 semi-synthetic cases, simulating realistic head shapes; the extraction of 138 morphometric descriptors per case for precise characterisation of head morphometry; the training and evaluation of machine learning models, including decision tree, random forest, and multilayer perceptron, for the automatic classification of six infant cranial deformities; and the demonstration of the clinical potential of integrating mobile 3D sensors, semi-synthetic data, and artificial intelligence for objective, reproducible, and accessible assessment of cranial deformities.

The article is structured as follows: Section 2 details the materials and methods, including data acquisition, processing, as well as semi-synthetic data generation; Section 3 presents the ML/DL models and training procedures; Section 4 discusses the results and compares them with previous studies; and Section 5 provides conclusions and outlines potential directions for future research.

## 2. Materials and Methods

### 2.1. PhotoMeDAS Application

PhotoMeDAS (version 1.7) is a photogrammetric application designed to assess cranial deformation, particularly in infants (https://photomedas.es). The tool was developed by the Photogrammetry and Laser Scanning Research Group (GIFLE) within the Department of Cartographic Engineering, Geodesy and Photogrammetry at the Universitat Politècnica de València (UPV) [23].

### 2.2. Smartphone Data Acquisition

The procedure begins by placing a coded cap and three orientation markers on the patient’s head. The data are then recorded using the PhotoMeDAS mobile application. After this, the processed results and the report can be accessed on the PhotoMeDAS website [32], which is currently available for clinical research use.

The Samsung Galaxy S22 Ultra [33] was selected as an optimal option thanks to its high-performance camera system, which provides sharp, high-resolution photos and videos, along with autofocus, stabilisation features, advanced recording functions, and a fast processor. A summary of these specifications is presented in Table 1. This smartphone offers a cost-effective solution for monitoring cranial growth or deformation, and it is fully compatible with the Android version of the PhotoMeDAS application. Its strong presence and proven reliability within the Android market were key factors in its selection for this study.

### 2.3. Initial Sample

A total of 60 three-dimensional point clouds of newborns’ heads were available, previously classified by specialists into six categories of cranial deformities: plagiocephaly (9 cases), brachycephaly (10 cases), dolichocephaly (11 cases), turricephaly or towering (13 cases), trigonocephaly (12 cases), and normocephaly (12 cases) [34,35].

### 2.4. Software

The processing of 3D data was carried out using CloudCompare, Visual Studio Code version 1.105.1 (user setup), and various Python libraries. For the development and implementation of the machine learning models—including random forest (RF), neural networks (multilayer perceptron, MLP), and decision trees (DTs), the libraries scikit-learn 1.2.2, NumPy 1.25.0, pandas 1.5.1, and Matplotlib 3.6.2 were employed. Additionally, the database was structured using Python 3.10.2 scripts with the os, shutil, and csv libraries, which allowed the automation of file organisation and system hierarchy, ensuring a reproducible and scalable workflow.

### 2.5. Hardware

The process was conducted on an MSI Stealth 15M laptop equipped with an Intel Core i7-1185G7 processor, 16 GB of RAM, and a 64-bit Windows 11 Home operating system.

### 2.6. Head Shapes

The head shape selection prioritised the diversity in head size, head shape and race, with ages spanning from 3 months to 3 years, leveraging males and females in the study. Data acquisition was conducted between November 2024 and April 2025 under controlled clinical conditions.

## 3. Workflow

**Figure 1:**
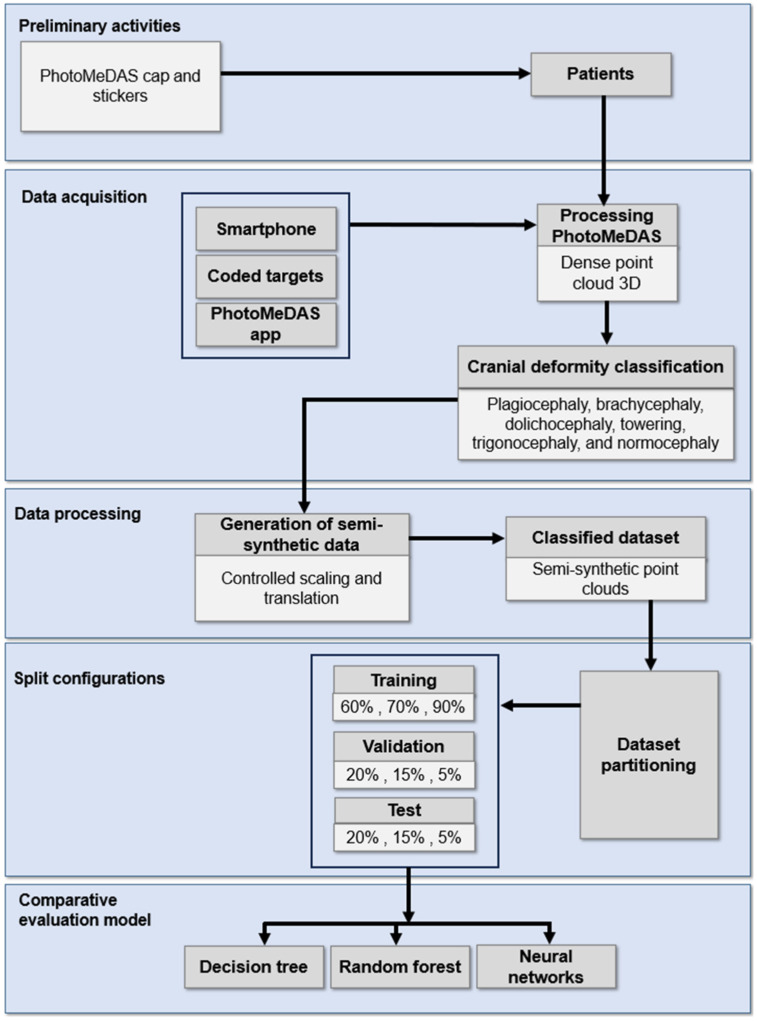
Workflow schema.

A detailed description of the methodology employed is presented in Figure 1. The workflow began with the acquisition of three-dimensional data from newborns’ skulls using a smartphone-based imaging system (Samsung S22 Ultra combined with a 3D scanning application) in a non-invasive manner. A total of 60 3D point clouds were collected and classified by specialists into six head shapes, including normocephaly and the following deformities: plagiocephaly, brachycephaly, dolichocephaly, turricephaly, and trigonocephaly. To expand and diversify the dataset, 3600 semi-synthetic 3D point clouds were generated through controlled geometric transformations, simulating plausible morphological variations. From each point cloud, 138 morphometric descriptors were extracted, representing the spatial characteristics of the skull by measuring distances from the centre of the cloud to the head surface. These descriptors were used to train and compare three machine learning models—DT, RF, and MLP—with the aim of enabling automatic classification of the six head shapes. Owing to its non-invasive approach, low cost, and rapid smartphone-based acquisition, the proposed workflow is well-suited for use in routine clinical environments as a complementary screening tool. It takes up to five minutes to carry out the smartphone scanning, and the same amount of time for processing (depending on the computational power of the server) to yield a full head anthropometric report.

### 3.1. Dataset and Acquisition

**Figure 2:**
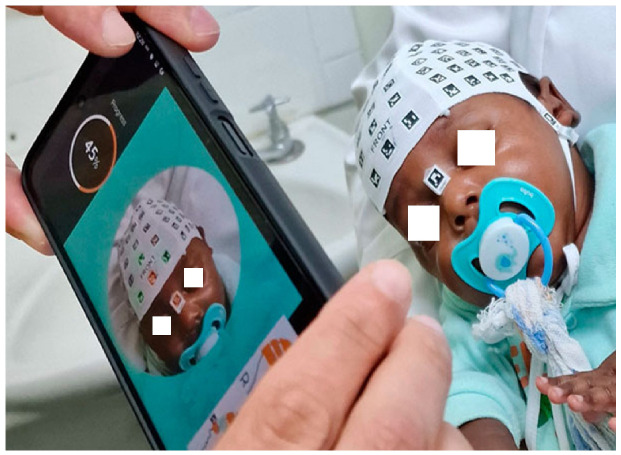
Data acquisition via the PhotoMeDAS mobile app.

This study was based on a three-dimensional (3D) dataset of infant 3D heads obtained after scanning via the PhotoMeDAS application (version 1.7) for Android and iOS devices (https://photomedas.es). Although the current data were acquired using a Samsung Galaxy S22 Ultra smartphone, the PhotoMeDAS application is compatible with other mid- to high-end smartphones. The data acquisition followed a standardised protocol, including the use of a fitted cap placed on the patient’s head to ensure morphological uniformity during scanning (Figure 2) [24].

**Figure 3:**
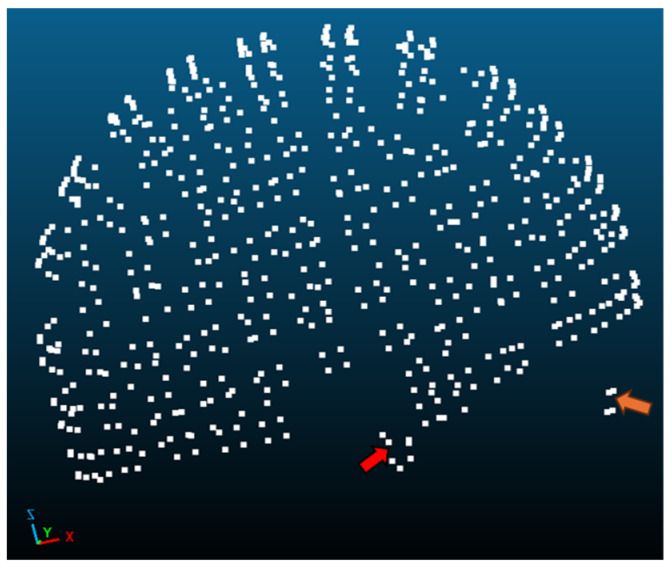
3D point cloud of the head obtained with the PhotoMeDAS app. The right arrow points to the sticker in the right preauricular area; the orange arrow points to the sticker between the eyes.

The PhotoMeDAS application generates 3D point clouds from multiple data captured at various angles around the head (Figure 3). The 3D coordinates generated by PhotoMeDAS are referenced to the three markers (front, left and right), allowing the automatic definition of both the origin and orientation of the spatial coordinate system, and establishing the primary orientation during processing. A detailed description of this reference system can be found in [23].

**Figure 4:**
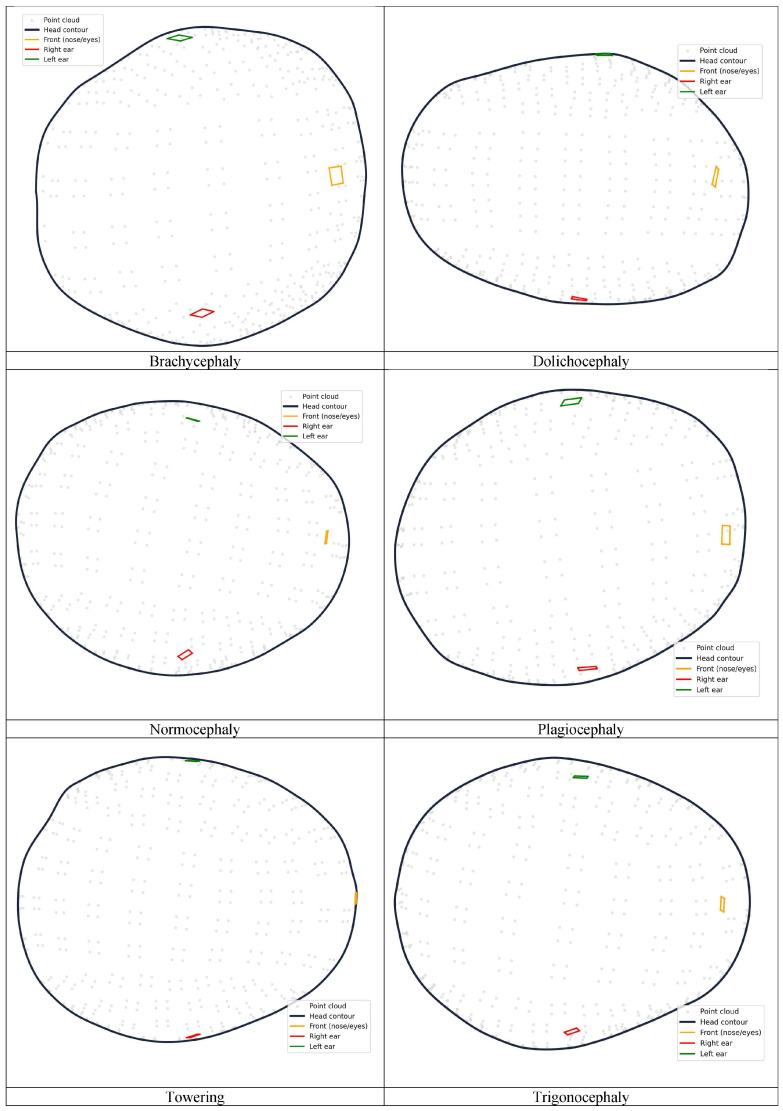
Top view visualisation of the point cloud for the six different head shapes. In the figure, the coded markers, which have four vertices, are colour-coded for clarity: red represents the right side, green represents the left side, and orange represents the frontal area corresponding to the nose above the eyes.

From each smartphone 3D scanning, a list of cranial anthropometric indices is reported, such as cranial asymmetry, cranial vault asymmetry index, perimeter 30, oblique cranial length ratio, cephalic index, towering index, metopic index, frontal angle and global index, among others. The formulation of each index can be found in [32]. In total, 60 3D point clouds of true infants were collected and classified into six morphological types (Figure 4). The classification was carried out in accordance with objective and normative criteria described in the literature, specifically following the “Normative ranges of anthropometric cranial indices and metopic suture closure during infancy” [36]: brachycephaly (10 cases), dolichocephaly (11 cases), normocephaly (five cases), plagiocephaly (nine cases), towering (13 cases), and trigonocephaly (12 cases). The 3D head models were classified according to the cranial anthropometric indices obtained from the PhotoMeDAS reports. The resulting database comprises 60 samples and is publicly available on the Zenodo repository [35].

After processing the data, the software automatically generates cranial anthropometric reports based on the determined 3D point cloud. The report presents ranges, 2D graphs and 3D models over time, illustrating cranial deformation [25].

### 3.2. Generation of Semi-Synthetic Data

To increase morphological diversity and improve the generalizability of the classification, 3600 semi-synthetic head point clouds were generated on the basis of an original dataset of 60 true patients previously classified with existing cranial deformities or not. The geometric transformations were designed to preserve the distinctive morphological features of each class while systematically introducing controlled variability. The resulting database comprises 3600 samples and is publicly available on the Zeno-do repository [37].

**Figure 5:**
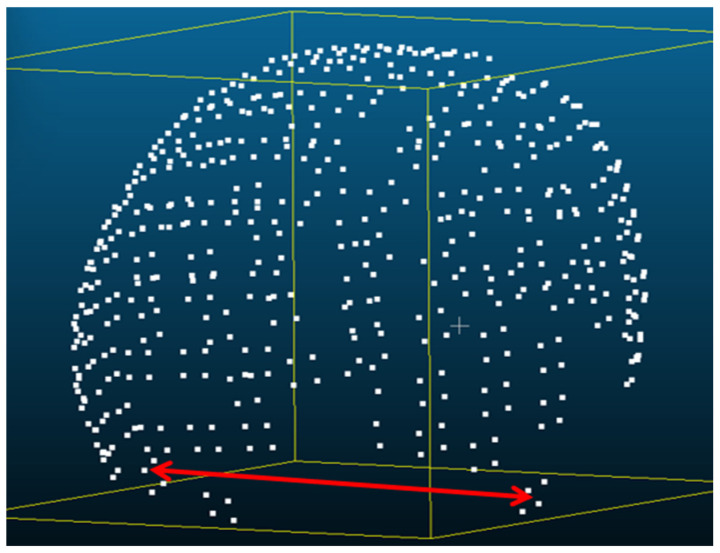
Point cloud with the red arrow representing the preauricular distance.

In the semi-synthetic data generation process, scaling parameters were established using the preauricular distance as the anatomical reference. This distance is defined as the linear measurement considering the centroid of both tragus markers L and R (Figure 5). A maximum variation margin of ±1.5 mm was set for this distance, in accordance with the precision limits documented in previous studies comparing the performance of PhotoMeDAS with that of reference 3D scanners [24]. That study reported mean errors ranging from ±1.0 to ±1.8 mm, depending on the device model used, thereby validating the choice of this threshold as representative of the expected variability under real clinical conditions.

**Figure 6:**
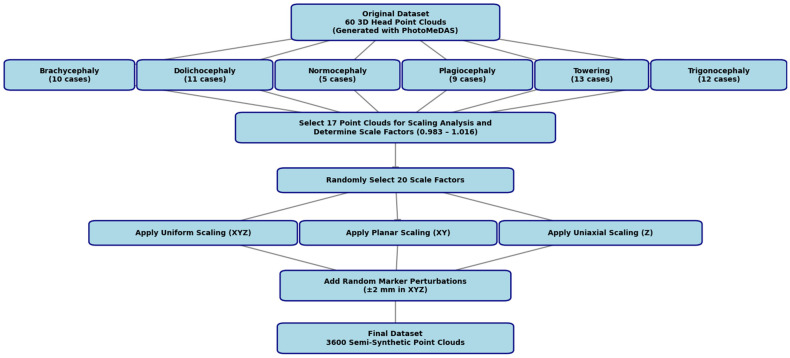
Semi-synthetic head 3D dataset generation process.

Figure 6 presents a flowchart of the stages involved in creating a dataset comprising 3600 semi-synthetic 3D point clouds.

The following procedure was followed (Figure 6): seventeen original 3D head point clouds were analysed to determine scaling factors that would maintain the preauricular distance within the ±1.5 mm margin. The resulting values ranged between 0.980 and 1.021, with an average range from 0.983 to 1.016. From this interval, twenty of the thirty-three possible factors were randomly selected, distributed evenly (e.g., 0.983, 0.984, …, 1.016), and applied as proportional transformations to the original point clouds.

The resulting geometric transformations of the initial 60 point clouds were performed under three configurations—applying 3D similarity transformations: (1) simultaneous scaling in X, Y, and Z; (2) scaling in the XY plane; and (3) uniaxial scaling in Z. This approach enabled the simulation of controlled morphological variations without altering diagnostic features, producing a robust and clinically realistic semi-synthetic dataset.

**Figure 7:**
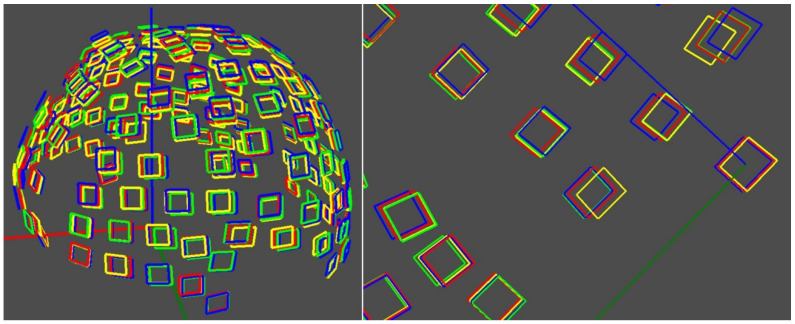
(**Left**) semi-synthetic scaling and superimposed 3D models. (**Right**) Noise applied to scaling across different axes and superimposed 3D models.

Additionally, random perturbations were applied to the anatomical marker coordinates, simulating variations inherent to the clinical process of cap placement. These perturbations, within a range of ±2 mm along the X, Y, and Z axes, generated subtle yet clinically relevant displacements (see Figure 7), thereby enhancing the dataset robustness against common positioning errors.

Each resulting dataset was described by Euclidean distances from the origin to the vertices on the cap (vid. Section 3.3), generating morphometric descriptors used as input variables for head deformity classification. The database was structured via Python scripts (libraries: os, shutil, and csv), automating file organisation and hierarchy to ensure a reproducible and scalable workflow.

### 3.3. Data Preprocessing

**Figure 8:**
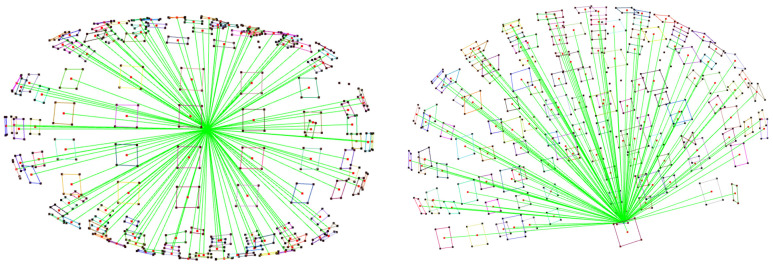
Top view (**left**) and side view (**right**) displaying the descriptors (distances) between each marker centroid and the origin of the coordinate reference system. The green lines highlight the descriptors, with a maximum of 138 per point cloud.

A dataset with a total of 3600 semi-synthetic point clouds was used. For each case, Euclidean distances were calculated between the origin of the coordinate reference system and each centroid of the coded markers placed on the elastic cap. In total, 540 vertices from 135 main markers were considered from the MC cap model (other cap models have a larger number of markers), together with 12 vertices from the three referencing stickers, resulting in 552 vertices in total. Since each marker has four vertices, the distances were averaged per marker to the centroid, resulting in 138 mean descriptors (distances) in the dataset (Figure 8).

The PhotoMeDAS application ideally requires the detection of 138 stickers on the patient’s head to generate the 3D point cloud; a minimum of 131 stickers is required. Table 2 displays the distribution of all calculated centroid distances (rows) by category (columns), reporting the number of distances considered per head deformity. This range of 131–138 descriptors (distances) was subsequently organised into input vectors for training the machine learning models, together with the categorical labels that were numerically encoded as follows:

1 = brachycephaly, 2 = dolichocephaly, 3 = towering, 4 = trigonocephaly, 5 = plagiocephaly, and 6 = normocephaly (Figure 4). This procedure enabled the construction of a dataset that was representative of the various cranial deformities under investigation.

The database was processed and restructured in Python via the pandas library. The data were subsequently split into training, validation, and test sets with different ratios (60% 20% 20%, 70% 15% 15% and 90% 5% 5%). This made it possible to evaluate how the size of the training set affected both the accuracy and the generalisation of the models.

### 3.4. Artificial Intelligence Models and Evaluation

Three machine learning approaches were applied to classify the severity of cranial deformities: decision trees (DTs), random forests (RFs), and multilayer perceptrons (MLPs) neural network. Each approach was trained, optimised, and evaluated to determine its capacity to generalise across datasets.

The choice of the three methods—decision tree (DT), random forest (RF), and multilayer perceptron (MLP)—was based on interpretability, sample size, and reproducibility. Although deep learning represents the state of the art, it requires more data than the 60 real cases available. To address this, 3600 semi-synthetic cases were generated through controlled transformations. DT and RF allow direct interpretation of the 138 morphometric descriptors, while MLP captures non-linear relationships without large datasets. Together, these methods provide a robust, clinically interpretable, and reproducible approach for the automatic classification of infant cranial deformities.

DT are supervised learning algorithms that model decision processes through hierarchical tree structures composed of nodes, branches, and leaves [38]. Each internal node represents a decision rule based on one feature, while terminal nodes (leaves) correspond to the predicted classes. The algorithm recursively partitions the dataset to minimise impurity, typically measured using the Gini index or entropy.

Several tree-building strategies exist, including ID3 (Iterative Dichotomiser 3), which uses information gain as a splitting criterion; C4.5, an improved version that introduces pruning and handles continuous variables; and CARTs (Classification and Regression Trees), which supports both classification and regression tasks using binary splits based on Gini impurity or mean squared error [38].

In this study, a CART-based implementation was adopted, and hyperparameter optimisation was performed using RandomisedSearchCV [39] to tune the maximum tree depth, the minimum number of samples required to split a node, and the minimum number of samples per leaf.

RF extends the DT paradigm through ensemble learning, combining multiple CART trees trained on random subsets of data and features [40]. This approach reduces overfitting and variance by averaging the predictions of individual trees, resulting in more stable and accurate outcomes.

In this research, the key parameters—the number of estimators (trees), the maximum depth, and the number of features considered at each split—were systematically optimised to enhance overall model performance.

MLP is a feedforward artificial neural network composed of interconnected layers of neurons capable of approximating complex non-linear relationships [41]. Each neuron applies an activation function—commonly the Rectified Linear Unit (ReLU) or hyperbolic tangent (tanh)—to introduce non-linearity into the model.

In this study, architectures with one to four hidden layers were evaluated, employing the Adam and SGD (Stochastic Gradient Descent) optimisers under both constant and adaptive learning rate strategies. To improve convergence, input data were standardised using StandardScaler. Hyperparameter optimisation was conducted using GridSearchCV to identify the most effective model configuration.

Training and validation were carried out via different dataset partitions (60% 20% 20%, 70% 15% 15% and 90% 5% 5%). The three machine learning approaches were evaluated in terms of accuracy, computational time, and generalisation capacity. This allowed us to analyse not only the performance of each algorithm but also the effect of training set size on the robustness of the results.

All the implementations were carried out in Python [42], using *scikit-learn* for model development and cross-validation [43]. Data handling and visualisation were performed with NumPy, pandas, and Matplotlib. Report generation and file management were performed with *FPDF* and standard Python libraries. Training was executed on a workstation equipped with an Intel Core i7 processor, 16 GB of RAM, and a dedicated GPU, which was especially used to optimise the performance of the neural networks.

## 4. Results

This section presents the quantitative results obtained from the three machine learning approaches, including comparisons of classification accuracy, computational efficiency, and the impact of hyperparameter selection. The outcomes are summarised in tables and followed by an analysis of the relative advantages and limitations of each method.

### 4.1. Machine Learning Approaches and Parameters

The three machine learning approaches used for classifying cranial deformities in infants, based on mobile photogrammetric data and 3D models (real and semi-synthetic), showed a strong dependence on hyperparameter settings and dataset partitioning. Additionally, the models’ performance was carefully evaluated using systematic hyperparameter searches (Table 3). For DT, a RandomizedSearchCV with 100 random iterations and 3-fold cross-validation yielded 300 training runs, allowing exploration of a wide range of tree depths and node constraints. For RF and MLP, GridSearchCV was employed, exploring 960 and 48 hyperparameter combinations, respectively, with 3-fold cross-validation, yielding 2880 and 144 total training iterations, respectively. This exhaustive search ensured that the reported results are reproducible and represent the best achievable configurations for the input data.

Under optimal configurations (Table 4), the DT, with a maximum depth of 19 and minimal restrictions on node size, struck a balance between complexity and fit. RF consistently outperformed single DT due to the advantages of ensemble learning. Neural networks, with two hidden layers (100 and 50 neurons) and the Adam optimiser, reached almost perfect accuracy across all dataset splits.

By contrast, less favourable configurations (Table 4) highlighted the susceptibility of certain methods to underfitting or poorly tuned parameters. Shallow DT (maximum depth = 3) substantially reduced predictive performance. RF, although more stable, was also weakened by shallow depth and larger leaf size requirements. Neural networks proved comparatively robust, though their performance declined when using overly simple architectures (e.g., a single hidden layer with four neurons) or less efficient optimisers such as SGD.

Hyperparameter Optimisation, Optimal configurations were identified for each model to maximise accuracy without excessively increasing the training time. For the DT, min_samples_split = 3, min_samples_leaf = 1, and max_depth = 19 were key for high performance. In RF, n_estimators between 50 and 100 are selected, with max_features set to ‘auto’, and the results are optimised. Neural networks benefit from architectures with two hidden layers (100 and 50 neurons) using ReLU activation, with the Adam optimiser accelerating convergence.

### 4.2. Precision, Recall and F1-Score

A detailed evaluation of precision, recall, and F1-score (Table 5) showed clear differences in the robustness of the models. DT were the least stable, with F1-scores falling to 0.51 in unfavourable settings, indicating high sensitivity to dataset partitioning and limited generalisation. RFs were more consistent, with worst-case F1-scores between 0.69 and 0.74, reflecting the benefits of ensemble learning. Neural networks were the most reliable; even under less favourable conditions, they maintained F1-scores above 0.90, highlighting their robustness for complex, high-dimensional data.

### 4.3. Computation Times

The total computation times for training, validation, and testing revealed significant differences between the models, as shown in Table 6.

DT was the most computationally efficient model, with total computation times ranging from 12.0 to 20.9 s. The RF algorithm required substantially longer times, ranging from 706.6 to 1014.2 s, due to the complexity of constructing multiple decision trees, which may limit its use in real-time applications. The MLP neural network exhibited intermediate times, between 200.3 and 233.6 s, reflecting a favourable balance between predictive accuracy and computational efficiency.

The high performance of the three machine learning approaches confirms the power of the descriptors to match the various shapes in the semi-synthetic data, which range from brachycephaly, normocephaly, to dolichocephaly, and plagiocephaly, without omitting uncommon shapes such as trigonocephaly and turricephaly. The 138 descriptors are able to learn geometric patterns from 3D point clouds, capturing small variations in head distances and shapes to distinguish deformities across the six categorical labels.

## 5. Discussion

This section presents a quantitative analysis of model performance, focusing on accuracy, F1-score, precision, recall, and computational times to provide an objective comparison of machine learning approaches for infant cranial deformity detection.

The results demonstrated that the selection of both the algorithm and its hyperparameters had a decisive impact on accuracy and computational efficiency in the detection of cranial deformities in infants, consistent with previous studies in craniofacial and paediatric artificial intelligence research [17,28,29].

The DT achieved precision of up to 0.98 under optimal conditions (Table 6), although its performance decreased substantially in less favourable configurations, with F1-scores dropping to as low as 0.51. This sensitivity to hyperparameter tuning and training set variability has been previously reported in classical machine learning approaches applied to biomedical data [39,43]. While DT offers fast inference, its instability limits its reliability for consistent clinical deployment.

In contrast, the RF showed greater robustness, reaching 100% accuracy in the best configurations and maintaining F1-scores between 0.67 and 0.74 in weaker scenarios. Its ensemble-based architecture mitigates overfitting and enhances generalisation [40,44], which is particularly advantageous for heterogeneous datasets derived from cranial morphology studies [23,24]. However, this robustness was associated with a high computational cost, with training times exceeding 1000 s, thereby limiting its applicability in real-time or mobile medical applications [26].

The MLP neural network provided the best overall balance between performance and efficiency. It consistently achieved F1-scores above 0.90, along with high precision and recall across all configurations, demonstrating strong generalisation capabilities, in agreement with recent studies employing deep and graph-based neural networks for craniofacial anomaly detection [45,46,47].

From a clinical perspective, these findings align with earlier evidence highlighting the importance of accurate and reproducible computational tools for cranial asymmetry assessment [13,14,16]. Moreover, advances in smartphone-based photogrammetry and synthetic data generation further reinforce the potential of lightweight neural models for 3D cranial shape analysis [23,24,27].

This performance compared favourably with previous studies based on deep learning applied to craniosynostosis. For example, the CS ResNet50 model achieved an overall test accuracy of 90.6% [48]. The sensitivity and accuracy were: 100% and 100% for metopic, 93.3% and 100% for sagittal, and 66.7% and 100% for unicoronal. This performance is comparable to that observed in the worst-case scenarios of the classical models evaluated in our study, specifically the DT and the RF, whose F1-score values ranged between 0.51 and 0.74. In contrast, the neural network-based model consistently overcomes these limitations, maintaining high sensitivity and F1-score values even under adverse data partitioning conditions.

In studies that use 3D data (e.g., [49,50]), exceptionally high-performance metrics were reported, with accuracies of up to 99.5% and sensitivities greater than 94%. However, these results depend on the use of specialised infrastructure, such as 3D stereophotography or computed tomography, which limits their applicability as large-scale screening tools. In contrast, our results demonstrate that models trained with more accessible data can achieve comparable metrics, particularly when neural architectures are used, thus reinforcing the feasibility of approaches oriented towards early screening in clinical contexts with limited resources.

The study presented in [29] achieved an accuracy of 90% in the classification of the surgical indication; however, it showed a reduced sensitivity in healthy cases (45%), mainly attributed to class imbalance. This phenomenon was also observed, although to a lesser extent, in our non-neural models, whose worst F1-score values decrease as the percentage of data allocated to training increases, reaching values of up to 0.51 in the 90% 5% 5% scheme. In contrast, the neural network showed greater stability against variations in the *data split*, suggesting better generalisation.

In addition, in accordance with what was reported in [51], where interobserver variability significantly impacted model performance on unseen data (75% accuracy), evaluating our worst-case results provided a more realistic estimate of system performance. The explicit inclusion of worst-case metrics allows us to demonstrate that, even under unfavourable conditions, the neural model maintains clinically acceptable performance, reinforcing its potential as a screening tool.

Overall, the results reveal a clear trade-off between speed, stability, and computational cost. The DT demonstrated fast processing but lower stability; the RF had more stability, although with a higher computational cost; and the MLP neural network represented the most balanced approach, combining high accuracy, robustness, and moderate computational demands. These characteristics make the MLP neural network the most promising option for clinical applications aimed at the early detection, screening, diagnosis aid, and monitoring of cranial deformities in infants, particularly when time is considered a limiting factor.

### Clinical Implications

Early detection of cranial deformities through smartphone-based scanning allows clinicians to quantify objectively head dimensions, expedite screening and improve diagnostic accuracy. The integration of artificial intelligence can further enhance the effectiveness of preliminary screening, particularly in regions with limited access to specialised studies, optimise resource allocation, prioritise patients requiring specialised care, and monitor the progression of cranial deformities. This approach is especially valuable in primary care settings, where it can serve as a complementary screening tool for patient assessment and follow-up.

## 6. Conclusions

This study evaluated the machine learning performance of DT, RF, and MLP neural network for helping to diagnose cranial deformities in infants using smartphone head scanning data through semi-synthetic 3D datasets. The inclusion of semi-synthetic data expanded the diversity and size of the initial point clouds, improving model generalisation and offering a more robust basis for evaluating performance across variable clinical scenarios.

The selection of machine learning approaches and the tuning of hyperparameters had a decisive influence on classification performance. Under optimal configurations, all models achieved near-perfect precision, recall, and F1-scores ≈ 1.00. However, performance varied considerably under less favourable conditions. The DT achieved precision and F1-scores of up to 0.98 in the best scenarios, but its performance declined sharply under suboptimal configurations, with F1-scores dropping to as low as 0.51, indicating high sensitivity to dataset partitioning and limited generalisation capacity.

In comparison, the RF demonstrated improved robustness, maintaining F1-scores between 0.69 and 0.74 in weaker configurations, reflecting the benefits of ensemble learning, although at a high computational cost. In contrast, the MLP neural network consistently achieved F1-scores above 0.90 across all configurations, demonstrating strong generalisation and stability. These findings highlight a clear trade-off between efficiency, robustness, and computational demand, positioning the MLP as the most balanced and clinically viable approach for fast screening of cranial deformities.

The analysis of computation times similarly revealed a trade-off between efficiency and overall classification performance. The DT remains the fastest, but also the least stable; the RF provides greater robustness at the expense of increased resource usage; and the MLP neural network occupies an intermediate position, requiring more computation time or processing power than the DT while offering an appropriate balance between performance and suitability for clinical workflows. From a sensor-based systems perspective, this study demonstrates the feasibility of leveraging optical sensors integrated into smartphones for 3D cranial data acquisition, supporting the development of accessible, non-invasive, and scalable screening solutions. Effective integration of this approach into clinical practice will require reductions in processing time and further improvements in robustness to achieve success rates close to 100%.

## Figures and Tables

**Table 1:** Samsung Galaxy S22 Ultra specifications.

Characteristic	Description
System	Operating System Android 12, Processor Exynos 2200 Octa-Core
Camera	12 MP, f/2.2, FF, FOV 120°, Sensor 1/2.55″, Pixel 1.4 µm, Maximum resolution 4000 × 3000 px
Connectivity	Mobile Network 5G, WIFI 802.11 a/b/g/n/ac, Bluetooth v5.0, NFC Yes
Display	Size 6.1″, Resolution 2340 × 1080 px.

Source: https://www.samsung.com/es/support/mobile-devices/check-out-the-new-camera-functions-of-the-galaxy-s22-series/ (accessed on 31 December 2025).

**Table 2:** Summary of descriptors per cranial deformity in the dataset.

Descriptors	Brachycephaly	Dolichocephaly	Normocephaly	Plagiocephaly	Towering	Trigonocephaly
138	180	60	180	180	180	300
137	0	60	0	0	120	60
136	120	180	120	60	180	120
135	180	120	0	0	180	0
134	0	120	0	180	120	180
133	60	120	0	120	0	0
132	60	0	0	0	0	0
131	0	0	0	0	0	60
Subtotal	600	660	300	540	780	720
Total	3600					

**Table 3:** Hyperparameter search configurations and total training iterations for evaluated machine learning models.

Machine Learning Approaches	Search Type	Hyperparameters Explored (Values)	Number of Combinations	Fold Cross-Validation	Total Training Runs
DT	RandomizedSearchCV	max_depth = [3,…,20], min_samples_split = [2,…,20], min_samples_leaf = [1,…,20]	100 random iterations	3	300
RF	GridSearchCv	n_estimators = [50,100,150,200], max_depth = [3,5,10,15,20], min_samples_split = [2,5,10,15], min_samples_leaf = [1,2,5,10], max_features = [‘auto’,’sqrt’,’log2’]	4 × 5 × 4 × 4 × 3 = 960	3	2880
MLP	GridSearchCv	hidden_layer_sizes = [(4,), (100,50), (100,100,50), (200,100,50), (200,200,100,50), (300,200,100,50)], activation = [‘relu’,’tanh’], solver = [‘adam’,’sgd’], learning_rate = [‘constant’,’adaptive’]	6 × 2 × 2 × 2 = 48	3	144

**Table 4:** Hyperparameter configurations of machine learning models under high- and low-performance settings.

Machine Learning Approaches	High-Performance Configuration	Low-Performance Configuration
DT	min_samples_split = 3	min_samples_split = 20
	min_samples_leaf = 1	min_samples_leaf = 18
	max_depth = 19	max_depth = 3
RF	n_estimators = 50–100	n_estimators = 50–200
	max_depth = 10	max_depth = 3
	max_features = auto	max_features = log2
	min_samples_leaf = 1	min_samples_leaf = 10
	min_samples_split = 2	min_samples_split = 2
MLP	activation = ReLU	activation = ReLU
	hidden_layers = (100,50)	hidden_layers = (4) or (100,50)
	learning_rate = constant	learning_rate = constant
	solver = Adam	solver = SGD/Adam

**Table 5:** Precision, recall, and F1-score under best and worst configurations for the different training, validation, and test ratios.

Training—Validation—Test	Model	Precision (Best)	Recall (Best)	F1-Score (Best)	Precision (Worst)	Recall (Worst)	F1-Score (Worst)
60%—20%—20%	DT	0.98	0.98	0.98	0.66	0.60	0.58
	RF	1.00	1.00	1.00	0.74	0.71	0.70
	MLP	1.00	1.00	1.00	0.95	0.91	0.93
70%—15%—15%	DT	0.98	0.98	0.98	0.65	0.58	0.53
	RF	1.00	1.00	1.00	0.71	0.70	0.69
	MLP	1.00	1.00	1.00	0.93	0.89	0.90
90%—5%—05%	DT	0.98	0.98	0.98	0.61	0.54	0.51
	RF	1.00	1.00	1.00	0.75	0.74	0.74
	MLP	1.00	1.00	1.00	0.92	0.90	0.90

**Table 6:** Total computation times (in seconds) of DT, RF, and MLP models for different dataset splits (train/validation/test).

Training—Validation—Test	DT	RF	MLP
60%—20%—20%	12.0 s	706.6 s	200.3 s
70%—15%—15%	13.3 s	794.9 s	215.9 s
90%—05%—05%	20.9 s	1014.2 s	233.6 s

## Data Availability

The data presented in this study are available on request from the group leader, José Luis Lerma.
